# Direct Composite Laminate Veneers: Three Case Reports

**DOI:** 10.5681/joddd.2013.019

**Published:** 2013-05-30

**Authors:** Bora Korkut, Funda Yanıkoğlu, Mahir Günday

**Affiliations:** ^1^Postgraduate Student, Department of Operative Dentistry, Marmara University, Faculty of Dentistry, Nişantaşı, Istanbul, Turkey; ^2^Professor, Department of Operative Dentistry, Marmara University, Faculty of Dentistry, Nişantaşı, Istanbul, Turkey; ^3^Professor, Department of Endodontics, Marmara University, Faculty of Dentistry, Nişantaşı, Istanbul, Turkey

**Keywords:** Composite restorations, dental esthetic, discoloration, fracture,, laminate, resin

## Abstract

Re-establishing a patient’s lost dental esthetic appearance is one of the most important topics for contemporary dentistry. New treatment materials and methods have been coming on the scene, day by day, in order to achieve such an aim. Most dentists prefer more conservative and aesthetic approaches, such as direct and indirect laminate veneer restorations, instead of full-ceramic crowns for anteriors where aesthetics is really important.

Laminate veneers are restorations which are envisioned to correct existing abnormalities, esthetic deficiencies and discolo-rations. Laminate veneer restorations may be processed in two different ways: direct or indirect. Direct laminate veneers have no need to be prepared in the laboratory and are based on the principle of application of a composite material directly to the prepared tooth surface in the dental clinic. Indirect laminate veneers may be produced from composite materials or ceramics, which are cemented to the tooth with an adhesive resin. In this case report, direct composite laminate veneer technique used for three patients with esthetic problems related to fractures, discolorations and an old prolapsed restoration, is described and six-month follow-ups are discussed. As a conclusion, direct laminate veneer restorations may be a treatment option for patients with the esthetic problems of anterior teeth in cases similar to those reported here.

## Introduction

Re-establishing a patient’s lost natural dental esthetics is among the important topics of today’s dentistry, in addition to function and fonation.^1^Color, shape, and structural and position abnormalities of anterior teeth might lead to important esthetic problems for patients.^2^ In order to solve such problems, the technique preferred frequently is to cover the teeth with dental crowns.^3^However, excessive preparations of teeth and damages to surrounding tissues, such as gingiva, are some disadvantages of crowns.^4^Therefore, in recent years, laminate veneer restorations, as a more esthetic and more conservative treatment option, have been used in dentistry.^12,4,5^

Laminate veneers are restorations which are envisioned to correct existing abnormalities, esthetic deficiencies and discolorations.^1,6^ Laminate veneer restorations have two different types: direct and indirect laminate veneers. Direct laminates are applied on prepared tooth surfaces with a composite resin material directly in the dental clinic. Absence of necessity for tooth preparation, low cost for patients compared with indirect techniques and other prosthetic approaches, reversibility of treatment and no need for an additional adhesive cementing system are some advantages of this technique.^7,8^ Intraoral polishing of direct laminate veneers is easy and any cracks or fractures on the restoration may be repaired intraorally^9^ and marginal adaptation is better than that of indirect laminate veneer restorations.^10^ However, the main disadvantages of direct laminate veneers are low resistance to wear, discoloration and fractures.^3,7,10^

Indirect laminate veneers with high resistance against attrition and fractures^10^ and discolorations^11^ have some advantages compared to direct laminate veneer restorations. However, long chair time, higher cost and use of an adhesive cementing system are the main disadvantages of indirect laminate veneer restorations.^2,3,12^

Every new material or method introduced to the field of dentistry aims to achieve esthetics and successful dental treatments with minimal invasiveness. Therefore, direct laminate veneer restorations have been developed for advanced esthetic problems of anterior teeth.^2,3^ Tooth discolorations, rotated teeth, coronal fractures, congenital or acquired malformations, diastemas, discolored restorations, palatally positioned teeth, absence of lateral incisors, abrasions and erosions are the main indications for direct laminate veneer restorations.^1-3^

In this case report, direct composite laminate veneer technique, used for three patients with esthetic problems related to fractures, discolorations and an old prolapsed restoration, is described and success in six-month follow-ups is discussed.

## Case report

### Case 1

A 37-year-old male patient with incisal edge fractures of maxillary central incisors, and therefore with esthetic complaints, referred to the faculty clinics. Old composite restorations, surrounded with secondary caries, were detected at the mesial edges of maxillary central incisors of the patient after clinical and radiographic examinations. Moreover, attritions involving enamel and also dentin tissues at the incisal edges of maxillary and mandibular central incisors, due to a possible bruxism problem, were noted (Figure 1a).

 Figure 1. Secondary carious lesions at mesial margins of upper central incisors (a). Cervical step preparations (b). Transparent matrix band and wedge application (c).

**a:**
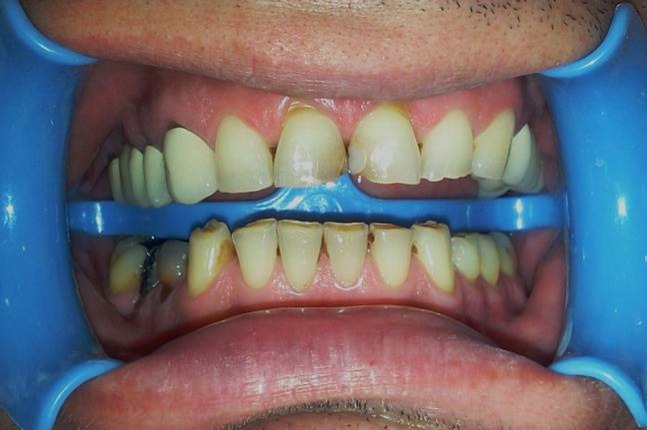

**b:**
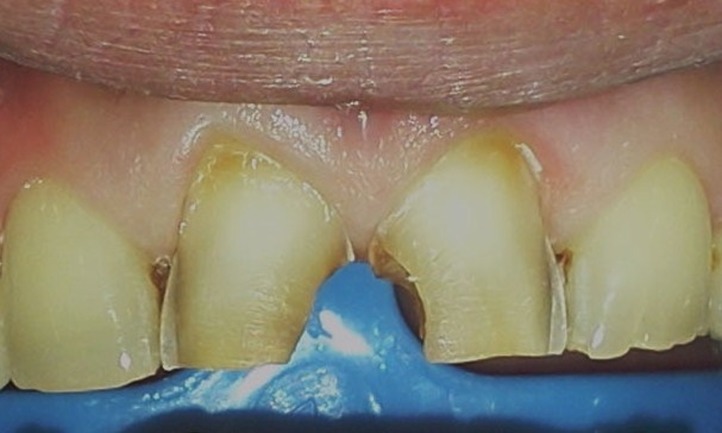

**c:**
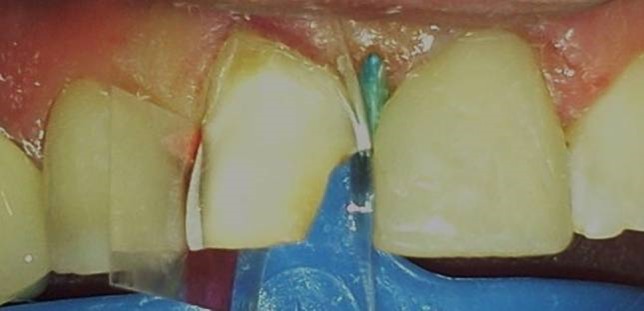

According to the patient’s history, the composite restorations were about five years old and the patient’s chief complaint was the unesthetic appearance of the restorations. He rejected ceramic laminate veneers or ceramic crowns as treatment options. The remaining tooth structures were adequate for both teeth for composite laminate veneer restorations. In spite of bruxism, direct composite laminate veneer restorations for both maxillary central incisors were considered, especially due to the need for the high compressive strength in this case.

Following local anesthetics for maxillary centrals, old restorations were removed with a high-speed handpiece (NSK Pana Air, Japan) and a round diamond bur (Acurata, Germany) under water cooling; 0.8-mm-deep walls were prepared on the labial surfaces of the teeth. Cervical borders of the preparations were arranged just at the same line of the gingiva by setting a cervical step without impinging on the natural gingival contour (Figure 1b).

As the cavity preparations were completed, shade was selected as A2 on the Vita scale.

For mesial and distal margins of the teeth, transparent matrix bands were cut, applied and fixed with appropriate wedges (Figure 1c). Then, 37% phosphoric acid (Etching Gel, Kerr, USA) was applied on the enamel surfaces for 15 seconds, rinsed with water spray for 20 seconds and dried slightly. One-bottle bonding agent (Adper Single Bond, 3M ESPE, USA) was applied in two layers on the prepared tooth surfaces by using a bonding brush and polymerized with a light-curing unit (Demi LED Light Curing System, 450 nm, Kerr, USA) for 20 seconds. In order to prevent any dark color reflection on the prepared surfaces, opaque A2 shade composite resin (Herculite XRV Ultra, Kerr, USA) was applied to the palatal sides and polymerized with a light-curing unit. Later, the cavities were filled with A2 shade of composite resin (Herculite XRV Ultra, Kerr, USA) incrementally and polymerized each time for 40 seconds.

For finishing and polishing procedures, first a yellow-banded knife-edge bur (Acurata, Germany) was used in a high-speed handpiece (NSK Pana Air, Japan). For advanced polishing, discs (Ultra Gloss Composite Polishing System, Axis, USA) in different dimensions were used from coarse to fine grits (Figure 2a).

Figure 2. Finishing and polishing (a). Six-month recall, no discolorations or disintegrations (b).

**a:**
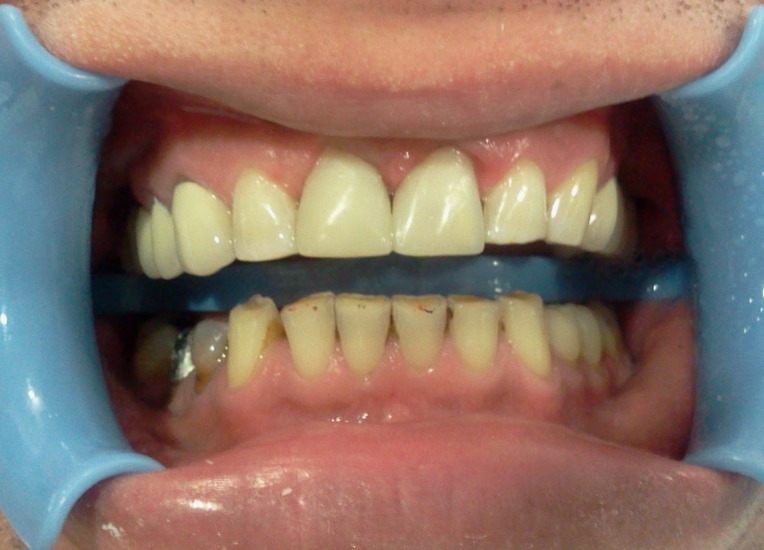

**b:**
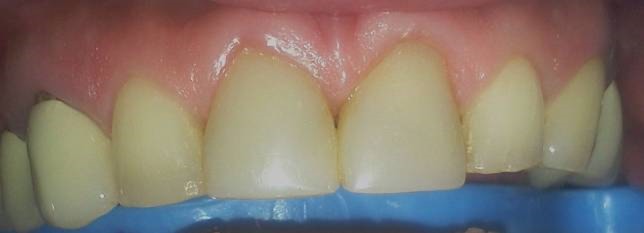

The patient was instructed in the oral hygiene and called at 6 months. At 6-month recall, no discolorations or disintegrations were detected (Figure 2b).

### Case 2

A 32-year-old female patient with esthetic complaints due to some stains on maxillary anteriors, presented to our faculty clinics. She also emphasized that her teeth were too small and she was not happy with this. After clinical and radiographic examinations, old composite resin restorations on teeth #11 and #21 and carious lesions on #12, #22 and #23 were detected (Figure 3a). The remaining tooth structures were adequate for composite laminate veneer restorations.

Figure 3. Upper incisors with carious lesions (a). Preparations with cervical steps (b,c).

**a:**
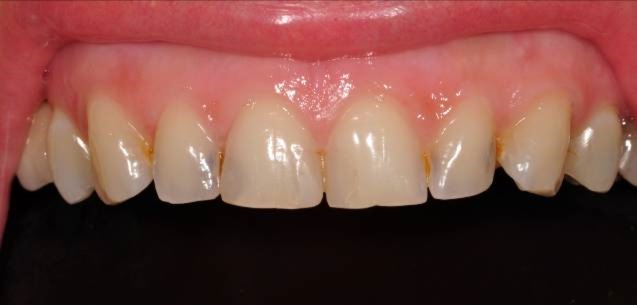

**b,c:**
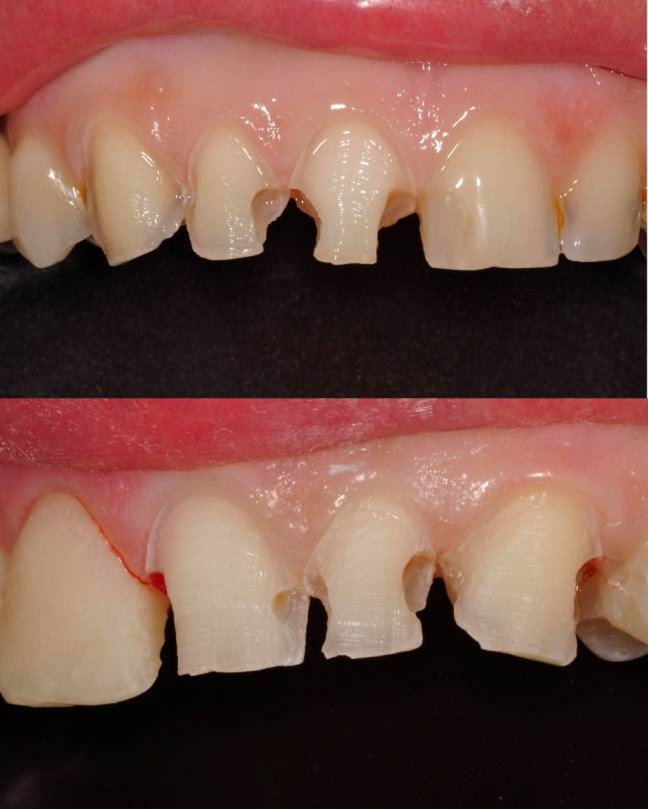

A2 shade was selected with GC shade guide. After the former restorations and the carious lesions were removed, direct composite restorations were considered treatment of choice due to small amount of remaining dental tissues of the teeth. Teeth #13 and #23 were also included in the treatment plan due to some stains and discolorations in order to achieve better esthetic results (Figure 3b,c). Laminate veneer preparations of labial surfaces were arranged to be limited within enamel borders. Preparation of cervical areas were arranged to be finished just short of the gingival margin.

As the preparations finished, translucent matrix bands, covering both the mesial and distal sides of the teeth, were applied to the cervical area and fixed with appropriate wedges; 37% phosphoric acid (Etching Gel, Kerr, USA) was applied for 15 seconds, rinsed with water spray for 20 seconds and slightly dried. A one-bottled bonding agent (Adper Single Bond, 3M ESPE, USA) was applied in two layers to prepared surfaces and polymerized with the light-curing unit for 20 seconds. An A2 dentin shade composite resin material (GC Gaenial, GC Corp., Tokyo, Japan) was applied palatally on the deep cavities of teeth #12, #11, #21, #22 and #23 and polymerized with the light-curing unit for 40 seconds. Then, A2 enamel shade of composite material (GC Gaenial, GC Corp., Tokyo, Japan) was applied gradually to the whole labial areas of the teeth and polymerized for 40 seconds every time (Figure 4a).

Figure 4. Composite restorations finished (a). Finishing (b,c). Polishing (d,e).

**a:**
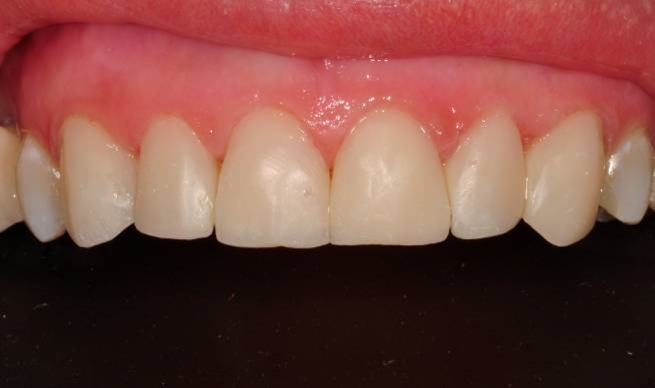

**b,c:**
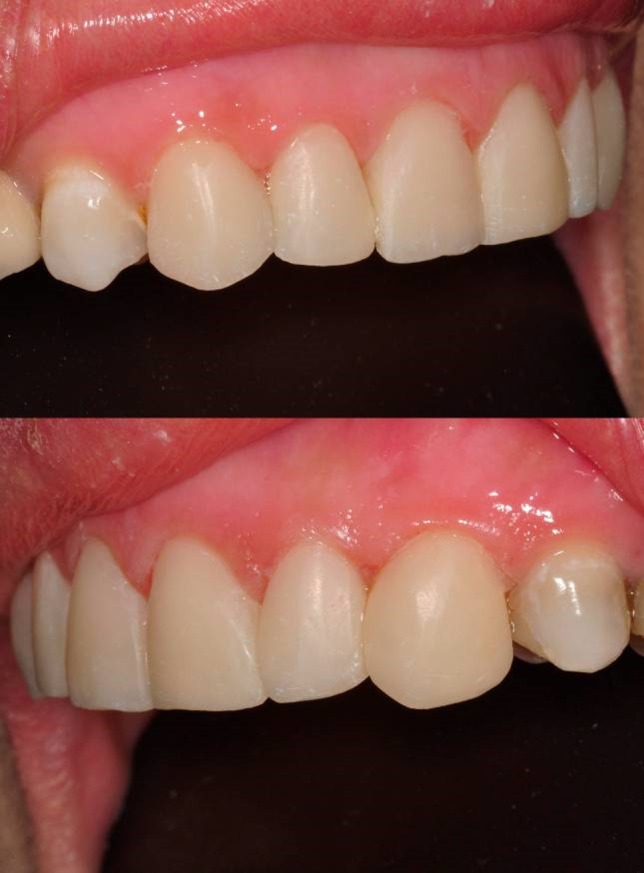

**d,e:**
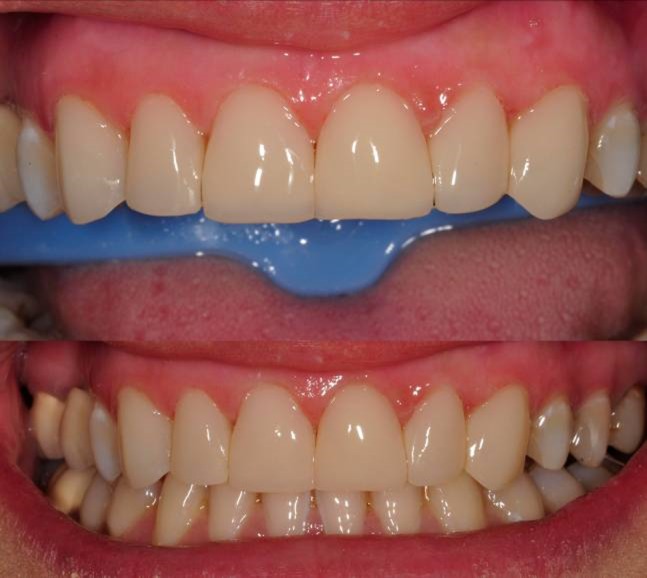

Finishing was achieved with a yellow banded diamond bur (Acurata, Germany) in a high-speed handpiece (Figure 4b,c). Then, polishing discs (Ultra Gloss Composite Polishing System, Axis, USA) were used in a low-speed handpiece (DURAtec 2068D, Germany) for fine polishing from coarse to fine grits (Figure 4d,e).

The patient was instructed in oral hygiene and was scheduled for control appointments once every 6 months. On the 6-month appointment, some discolorations on the contact areas of the teeth were detected (Figure 5a). However, the patient was very pleased with the current esthetic situation of her teeth so she wanted her premolars being treated with the same method. After clinical examination, teeth #14, #24 and #25 were considered for treatment with direct restorations (Figure 5b,c). As the restorations finished (Figure 5d), the patient was asked to use dental floss every day at contact areas.

Figure 5. Six-month recall; some discolorations at contact areas (a). Additional restorations to teeth #14, #24 and #25 (b,c). Composite restorations finished (d).

**a:**
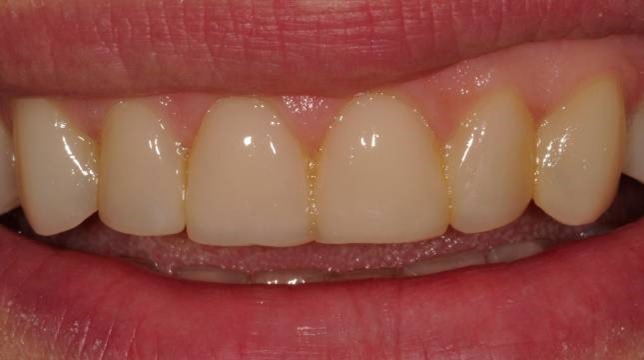

**b,c:**
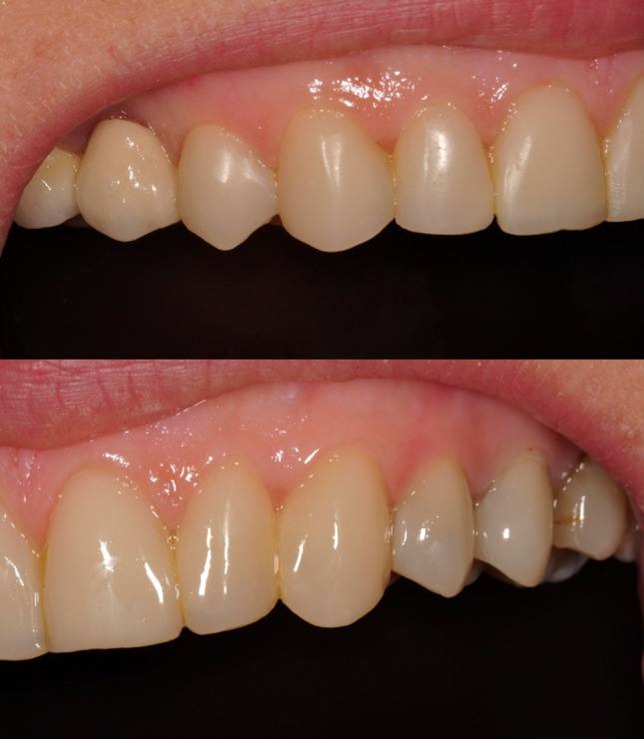

**d:**
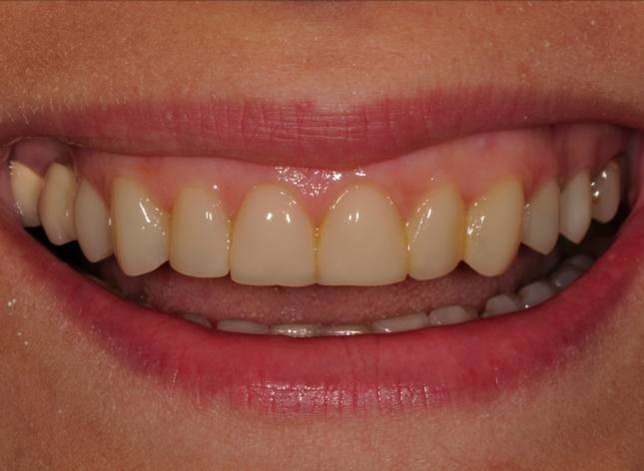

### Case 3

A 45-year-old female with esthetic complaints due to a prolapsed ceramic laminate veneer restoration on maxillary left central incisor referred to the faculty clinics. The patient reported that the ceramic laminate veneer restorations were nearly 1 year old and only the prolapsed one had caused problem. Moreover, the prolapsed restoration was lost. Carious lesions were detected on the mesial and labial surfaces of maxillary left central incisor clinically (Figure 6a). No sensitivities to percussion were detected horizontally and vertically. In radiographic examination, a previous endodontic treatment was detected and no pathologies were evident in the periapical area. Although there was a large amount of lost tooth structure, direct composite laminate veneer restoration was considered in order to complete the treatment as soon as possible as the patient demanded.

Figure 6. Prolapsed restoration of tooth #21 (a). Cervical step preparations (b).

**a:**
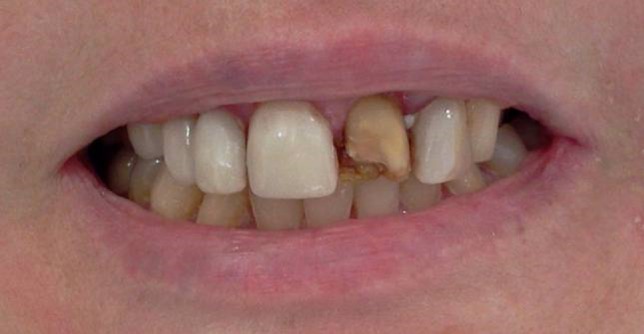

**b:**
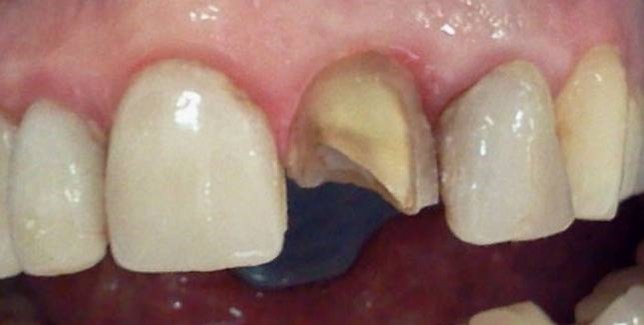

First, infected tooth structures were removed with a low-speed handpiece (DURAtec 2068D, Germany) and a stainless steel round bur (Acurata, Germany). Cervical borders of the preparation were arranged just at the same line with the gingiva (Figure 6b).

A2 shade was selected using Vita guide, corresponding with the laminate veneer on tooth #11. Translucent matrix bands were fixed on the mesial and distal contact areas with appropriate wedges; 37% phosphoric acid (Etching Gel, Kerr, USA) was applied for 15 seconds, rinsed for 20 seconds and dried slightly. Then, the bonding agent (Adper Single Bond, 3M ESPE, USA) was applied in two layers and polymerized for 20 seconds with the light-curing unit (Demi LED Light Curing System 450 nm, Kerr, USA). Opaque D2 shade composite resin (Herculite XRV Ultra, Kerr, USA) was applied on the mesial area where less tooth structure remained. Subsequently, A2 shade composite resin (Herculite XRV Ultra, Kerr, USA) was applied gradually to the entire prepared surfaces of teeth and polymerized with a light-curing unit for 40 seconds every time.

Finishing and polishing processes were carried out with a yellow-banded diamond bur (Acurata, Germany), immediately followed by polishing discs (Ultra Gloss Composite Polishing System, Axis, USA). The polishing discs were used in a low-speed handpiece (DURAtec 2068D, Germany) from coarse to fine grits (Figure 7a,b).

Figure 7. Before treatment (a), and after finishing and polishing (b). Six-month recall, no discolorations or disintegrations (c).

**a,b:**
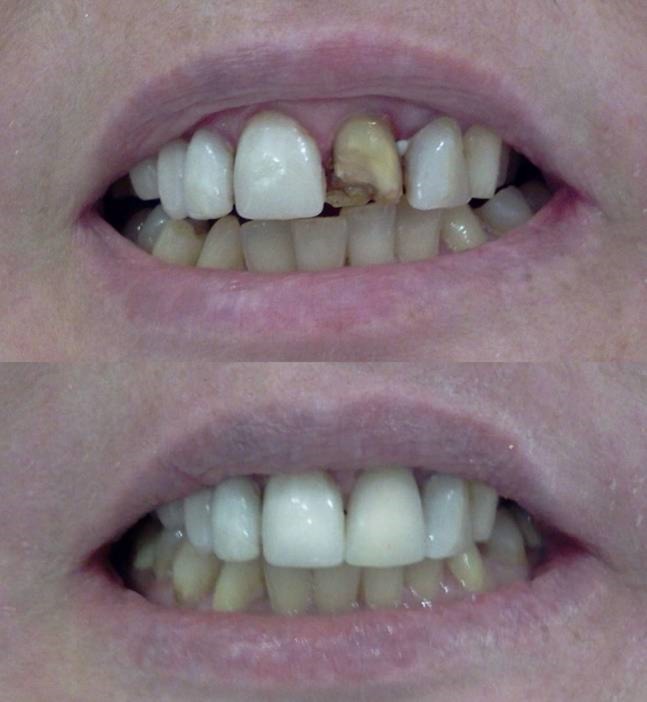

**c:**
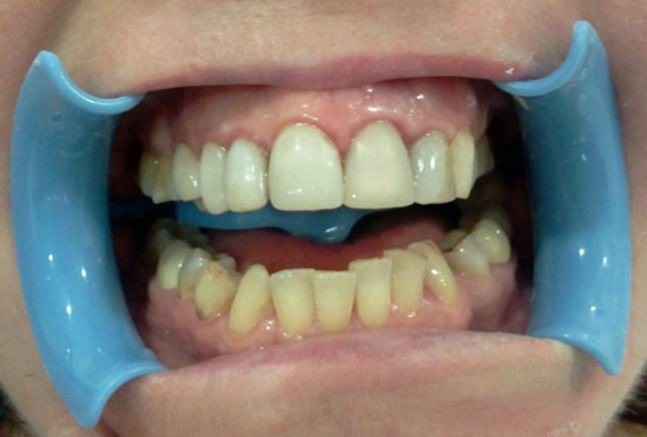

The patient was instructed in the oral hygiene and recall visits were scheduled every 6 months. No discolorations or disintegrations were detected at six-month recall (Figure 7c).

## Discussion

Direct and indirect laminate veneers, as esthetic procedures, have become treatment alternatives for patients with esthetic problems of anterior teeth in recent years.^2,6^In deciding between those two treatment options, the cost, social and time factors have to be considered.^2^Although ceramic laminate veneer restorations have some advantages like color stability and high resistance against abrasion, they have also some disadvantages, including high cost and long chair time.^2,3,12^ Moreover, they have some problems such as necessity of an additional adhesive cement. In addition, wrong indications, dentist–technician coordination problems during shade harmonization, inability to mask the underneath discolored dental tissue due to the low preparation depth, especially at the cervical area, long chair time for repairing simple fractures and simple inattentions during cementation are still important subjects waiting for solutions.^1,12-15^,Composite resins correct existing deficiencies, increase the physical properties and are now more esthetic options instead of laminate veneer applications.^1^Additionally, today’s dentistry requires more conservative treatment options.^12^Therefore, composite laminate veneer restorations, which require minimal removal of tooth structure, are one of the best treatment choices.^2,3,12,16^ With the advantages such as only one appointment for the whole treatment time, very low costs compared with the ceramics and no need for long laboratory procedures, direct composite laminate veneers are more popular in today’s dentistry.^1^

However, direct composite laminate restorations have still less resistance against abrasions and fractures compared with indirect composite laminate veneers and ceramic laminates.^2,8,10,12^Furthermore, indirect composite laminate veneer restorations due to polymerization outside of the oral cavity, and ceramic laminate veneers due to better color stability because of being less affected by the fluids of the oral cavity, are superior to direct composite laminate veneers.^2,10,17^

The most preferred treatment method for anterior teeth with esthetic problems is laminate veneer restorations;^6^ however, the condition in which the direct, indirect composite resin and indirect ceramic laminate veneers are chosen is very important for the success of the treatment. The dentist has to make the decision after a complete review and a correct indication after proper clinical examination. The dentist should also analyze the patient’s socioeconomic status, esthetic expectations and oral hygiene conditions completely.^1,2^

In case 1, in order to establish both functional and esthetic integrity, laminate veneers including the incisal edges were considered. In addition, considering more resistance and compressive strength than ceramics and similar abrasion rates compared with natural tooth structures, direct composite laminate veneer restorations were considered. Full-ceramic crown restorations were not considered because they result in excessive tooth structure loss.

In case 2, the discolorations on the contact areas on the 6-month recall can be related to the insufficient dental hygiene and no use of dental floss. These were the discolorations which can be easily removed by slight polishing. The patient was also instructed in the use of dental floss so that she would not have any problems again.

In case 3, there was a large amount of lost tooth structure. However, the patient wanted the treatment to be completed as soon as possible; therefore, direct composite laminate veneer restorations were considered instead of ceramic laminate veneers or full-ceramic crowns.

According to these case reports, although there are still some disadvantages, especially discolorations and fragility, with the development of new composite resins, direct laminate veneer restorations can be a treatment option for patients with esthetic problems of anterior teeth, when applied judiciously with good patient hygiene motivation.
